# Research on load clustering algorithm based on variational autoencoder and hierarchical clustering

**DOI:** 10.1371/journal.pone.0303977

**Published:** 2024-06-13

**Authors:** Miaozhuang Cai, Yin Zheng, Zhengyang Peng, Chunyan Huang, Haoxia Jiang

**Affiliations:** 1 Guangzhou Power Supply Bureau, Guangdong Power Grid Company, Guangzhou, China; 2 Guangzhou Benliu Power Technology Company, Guangzhou, China; Instituto Tecnologico de Aeronautica, BRAZIL

## Abstract

Time series data complexity presents new challenges in clustering analysis across fields such as electricity, energy, industry, and finance. Despite advances in representation learning and clustering with Variational Autoencoders (VAE) based deep learning techniques, issues like the absence of discriminative power in feature representation, the disconnect between instance reconstruction and clustering objectives, and scalability challenges with large datasets persist. This paper introduces a novel deep time series clustering approach integrating VAE with metric learning. It leverages a VAE based on Gated Recurrent Units for temporal feature extraction, incorporates metric learning for joint optimization of latent space representation, and employs the sum of log likelihoods as the clustering merging criterion, markedly improving clustering accuracy and interpretability. Experimental findings demonstrate a 27.16% improvement in average clustering accuracy and a 47.15% increase in speed on industrial load data. This study offers novel insights and tools for the thorough analysis and application of time series data, with further exploration of VAE’s potential in time series clustering anticipated in future research.

## 1. Introduction

Time series data are widely present in various fields of science and engineering, including electric load, energy monitoring, industrial intelligent diagnosis, and financial data [1–4]. The continuous growth of such data presents unparalleled challenges to the field of clustering research. Unlike static data, the characteristics of time series data are characterized by inherent multi-scale temporal dependencies that reflect the data’s dynamic changes, including both long-term and short-term pattern variations [5, 6]. This unique temporal characteristic renders most traditional clustering algorithms unsuitable for time series data, as they often fail to capture the dynamic essence of such data. Consequently, the development of new clustering methodologies that accommodate the distinct characteristics of time series data has emerged as a critical area of research [7].

In recent years, deep learning techniques have demonstrated significant efficacy in the domain of representation learning and clustering for time series data [8]. Compared to deep clustering methods designed specifically for static data, deep time series clustering algorithms place a special emphasis on modeling temporal dependencies [9]. These methods utilize the capability of autoencoders, combined with the non-linear characteristics of neural networks, to efficiently reduce data dimensionality and extract latent features of samples, thereby providing optimized data representations suitable for subsequent clustering [10, 11]. The Deep Temporal Clustering (DTC) framework by Madiraju, which integrates autoencoders with a specific temporal clustering layer, aims to minimize the Kullback-Leibler (KL) divergence between predicted and target distributions, thereby learning clustering assignments [12]. Furthermore, Ma et al. have combined k-means clustering with temporal reconstruction within a sequence-to-sequence (seq2seq) model, forming cluster-specific representations and constructing clustering structures under the guidance of a k-means objective [13]. Moreover, Avi has introduced a novel deep clustering algorithm employing a Variational Autoencoder (VAE) featuring a multi-encoder-decoder architecture to facilitate learning latent representations towards a more efficient spatial configuration [14]. Zhong’s approach, a deep temporal contrastive clustering method, learns representations via two similar autoencoders and combines a k-means objective to optimize the clustering distribution [15].

Although recent advancements have been notable in the field of deep time series clustering via generative models, existing methods continue to confront a number of critical challenges. First, numerous algorithms that rely on instance reconstruction or clustering distribution for representation learning fail to fully capitalize on the differences between samples, resulting in features that lack discriminative power, consequently diminishing the quality and practicality of clustering. Additionally, these methods frequently overlook the crucial interaction between reconstruction and clustering objectives, thereby leading to the degradation of the generative model and compromising training stability. Second, these algorithms often neglect the intrinsic hierarchical structures and detailed features of time series data, thereby restricting the interpretability of the clustering outcomes. Lastly, these algorithms encounter challenges related to scalability and computational efficiency in processing large datasets, and the absence of effective stopping criteria complicates the determination of the optimal cluster count.

In response to the aforementioned challenges, the present study proposes a novel deep time series clustering method that uniquely integrates metric learning with a hierarchical clustering strategy within the domain of deep time series clustering. This method encompasses four key components: (1) Utilizing a VAE based on gated neural networks to extract the temporal features of time series data specifically; (2) Enhancing discriminability in the latent space via metric learning and securing joint optimization of reconstruction loss, KL distribution, and metric learning loss to boost clustering accuracy; (3) Designing an output structure in alignment with agglomerative hierarchical clustering to enhance the interpretability and the ability to analyze details of clustering results; (4) Introducing a clustering merging criterion based on reduced log-likelihood along with effective stopping criteria to minimize computational costs and maximize the practicality of the algorithm.

The contributions of this study are summarized as follows: Implementing a learning mechanism specifically tailored to the features of time series data; Introducing improvements to the loss function to diminish reliance on unsupervised learning and achieve a balance between reconstruction and regularization losses; Enhancing the representational precision of clustering through metric learning; Providing detailed clustering outputs in alignment with hierarchical structures, and establishing reasonable clustering merging and stopping criteria to minimize the computational demands of hierarchical clustering. Lastly, validating the method’s superiority and efficiency through application to large-scale industrial load data.

The paper is structured as follows: Section II introduces related work; Section III details the proposed algorithm; Section IV showcases the algorithm’s test cases and results; finally, Section V summarizes the conclusions and discusses future research directions.

## 2. Related work and basic algorithms

In the following text, we discuss the related work in the field of deep time series clustering methods and the basic algorithms addressed in this paper.

### 2.1. Deep time series clustering algorithm

Time series clustering remains an important research topic in time series analysis [16]. Initially, we review traditional time series clustering methods, distinguishing between methods based on raw data versus those based on features. Subsequently, we proceed to review the latest advancements in deep time series clustering methods.

Methods based on raw data, also termed shape-based methods, directly employ raw time series data. A typical strategy involves relying on improved distance or similarity metrics, often in combination with traditional clustering algorithms [7]. For example, Suh et al. combined k-means with hierarchical clustering using dynamic time warping to achieve clustering [17]. Li et al. proposed a time series clustering method based on complex networks, which employs the density peak algorithm to transform time series into state sequences and calculate similarity, followed by clustering using community detection techniques [18]. Although these methods can capture local similarities in time series, they do not fully account for the global structure of the data [19].

Feature-based approaches prioritize transforming raw time series into low-dimensional feature representations to achieve more efficient clustering. Key advantages of these approaches include minimizing noise interference on clustering outcomes and reducing data dimensionality [7]. For instance, Afzal et al. extracted low-dimensional features using Independent Component Analysis (ICA) and utilized an enhanced K-means algorithm for clustering [20]. Zakaria et al. introduced the shapelets concept, identifying key local patterns within time series [21], while Ruan et al. simplified time series into symbolic sequences with Symbolic Aggregate Approximation (SAX), mitigating the stringent constraints on time series trends [22]. However, relying solely on features extracted through traditional methods for clustering does not adequately capture all the essential attributes of time series [19].

Advancements in deep learning techniques have significantly propelled developments in time series clustering technology. Training neural networks to learn unique data feature representations enables the automatic division of data into disjoint groups with minimal manual intervention [23]. Ienco proposed a semi-supervised (constrained) deep embedding time series clustering framework, utilizing knowledge-based supervision and modeling with Gated Recurrent Units (GRU) aimed at explicitly managing the temporal dimensions associated with multivariate time series data [24]. Furthermore, Ienco introduced an unsupervised clustering method for time series that employs a recurrent autoencoder integrated with attention and gating mechanisms for effective data embedding [25]. Kim introduced a deep bidirectional similarity learning model for clustering, which flexibly adjusts filter and pooling sizes based on data volume [26]. Xu developed a Recursive Graph Variational Autoencoder (RGVAE) deep clustering model that transforms time series data into recursive graphs for feature extraction, dimension reduction, and normalization of data distribution through a variational autoencoder, combining autoencoder reconstruction and clustering losses for clustering outcomes [27]. Despite significant progress in deep clustering methods, several challenges persist. Many algorithms rely on learning from instance reconstruction or clustering distribution yet fail to thoroughly explore inter-sample differences, leading to insufficiently discriminative features and affecting the quality and practicality of clustering outcomes. Moreover, existing methods often overlook the intrinsic hierarchical structure and detailed features of time series data, thereby limiting the interpretability of the clustering outcomes. Lastly, these algorithms face scalability and computational efficiency challenges when processing large datasets and typically lack effective stopping criteria, thus complicating the determination of the optimal number of clusters and increasing computational costs.

### 2.2. Basic algorithms

#### 2.2.1. Gaussian mixture models for clustering

The Expectation Maximization algorithm (EM) is a general framework for applying the K-means iterative structure to general distributions, and the EM algorithm is often used with Gaussian distributions. An intuitive difference between the EM algorithm and K-means is that while K-means defines the distance to the center point by drawing a circle around it, the EM algorithm uses a general ellipse [5].

When EM algorithm is applied to Gaussian framework, it uses Gaussian Mixture Model (GMM). The GMM consists of an arbitrary number of superimposed Gaussians. The probability distribution equation of GMM is as follows [28].

$$\begin{array}{cc} p(x) & =\overset{K}{\underset{i=1}{\sum }}\phi_{i}N(x|\;\;\mu_{i},\sum_{i})\\ & \overset{K}{\underset{i=1}{\sum }}\phi_{i}=1\\ & 1>\phi_{i}\;\forall 0 \end{array}$$ (1)

Where *K* is the number of Gaussians used to fit the dataset and *φ*_*i*_ is the relative weight assigned to this Gaussians. To draw a random sample from this distribution is equivalent to first drawing a sample from {1,…, K} distribution and then use that number to decide which (*μ*_*i*_, *Σ*_*i*_) to sample from. Instead of fitting an arbitrary dataset with a single Gaussian, GMM can be fitted with at least the same accuracy.

#### 2.2.2. Hierarchical agglomerative cluster

The agglomerative hierarchical clustering algorithm treats all data points in *X* as clusters and aims to iteratively merge the two clusters closest to each other based on the distance measure *d*. There are three main types of cohesive clustering, which differ in how the differences between groups of points are defined. They are single link, full link and average link number [7].

Single-linked clustering attempts to merge the two clusters with the shortest distance between the closest members in each cluster. The distance between two clusters *i* and *j* is defined as:

$$d_{SL}(i,j)={\text{min}}_{\left[a\in i,b\in j\right]}\;d_{a,b}$$ (2)

Single chaining emphasizes that close points must be in the same cluster. However, it can create large and unintuitive clusters that extend across hyperspace, and seemingly unrelated points become related due to long chains of relationships.

Full chaining takes the opposite approach and requires the least similar points in the two clusters to be closer to each other than any other pair of clusters to be merged. The distance between two clusters *i* and *j* is defined as:

$$d_{CL}(i,j)={\text{max}}_{a\in i,b\in j}\;d_{a,b}$$ (3)

Full links tend to create conservative clusters that don’t risk hyperspace.

Average links can be seen as an intermediate distance measure that measures the distance between cluster centers:

$$d_{avg}(i,j)=\frac{1}{n_{i}n_{j}}\sum_{a\in i}\sum_{b\in j}\;d_{a,b}$$ (4)

An efficient way to store and express hierarchical clustering is to use a link matrix, which is an (*n*-1)×3 matrix if the data set contains n data points. The first two columns indicate which clusters are merged, and the third column contains the distance between the two clusters based on the selected link. If the number of clusters in the first or second column is equal to or less than *n*, it means that data points with that index are merged, and if the number of clusters is greater than *n*, it means that a cluster consisting of two or more data points is merged with another cluster. If the cluster number is *i*>*n*, the cluster is defined as the merge found on rows *i*-*n* in the link matrix.

#### 2.2.3. Variational autoencoder

**Fig 1:**
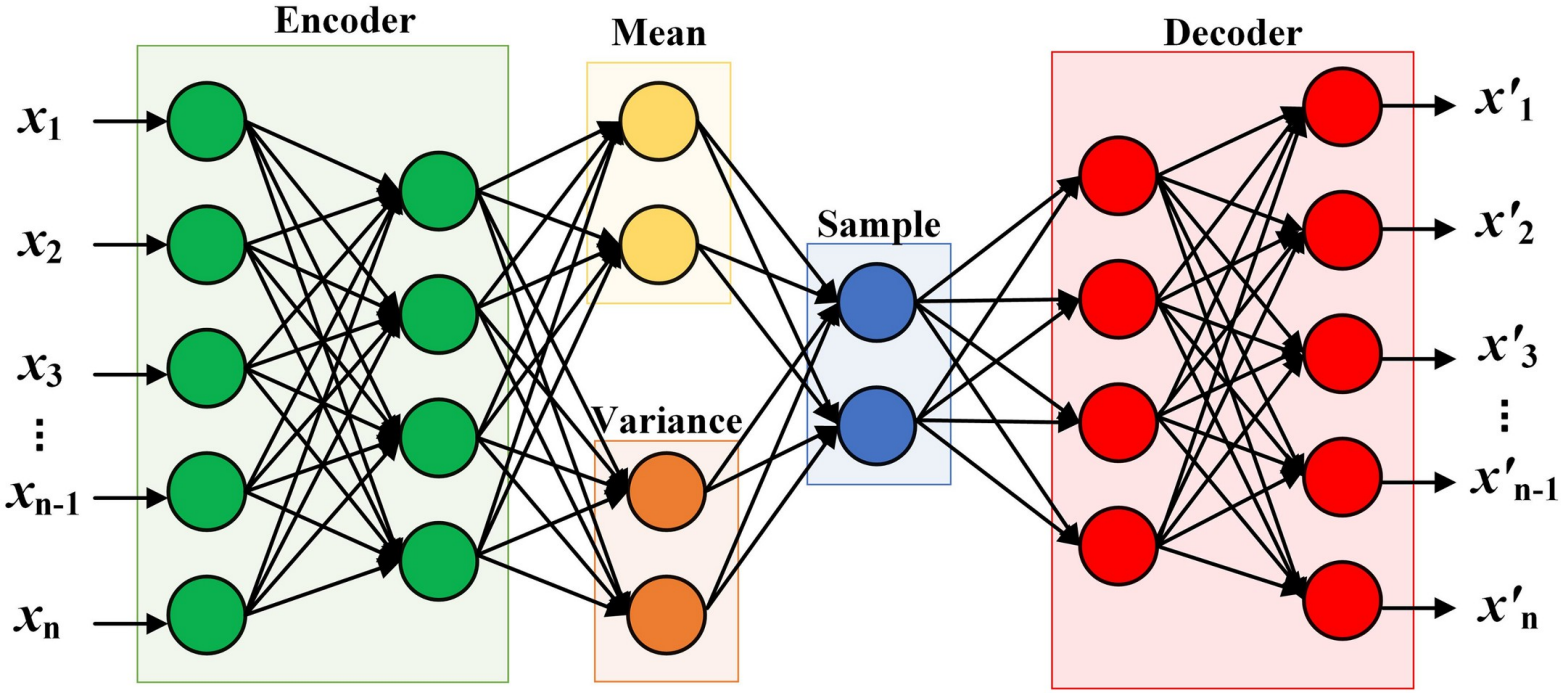
Typical variational autoencoder model structure.

Variational autoencoders (VAE) have received a lot of attention since their introduction in 2014, and their architecture is shown in Fig 1. VAE differs from conventional autoencoders in two ways: 1) It adds an extra term to its loss function, the KL divergence. KL divergence forces the potential representation to follow a certain distribution, usually a Gaussian distribution. When a sample is generated from a normal distribution and fed to a decoder, KL divergence ensures that the sample is not too far removed from any earlier points. KL divergence is not required to be included, but is usually included to make the network a well-functioning generative network. 2) It introduces noise into the potential space, making the potential representation slightly different each time the input passes through the network, greatly improving the capability of the autoencoder framework [9].

## 3. Proposed algorithm

**Fig 2:**
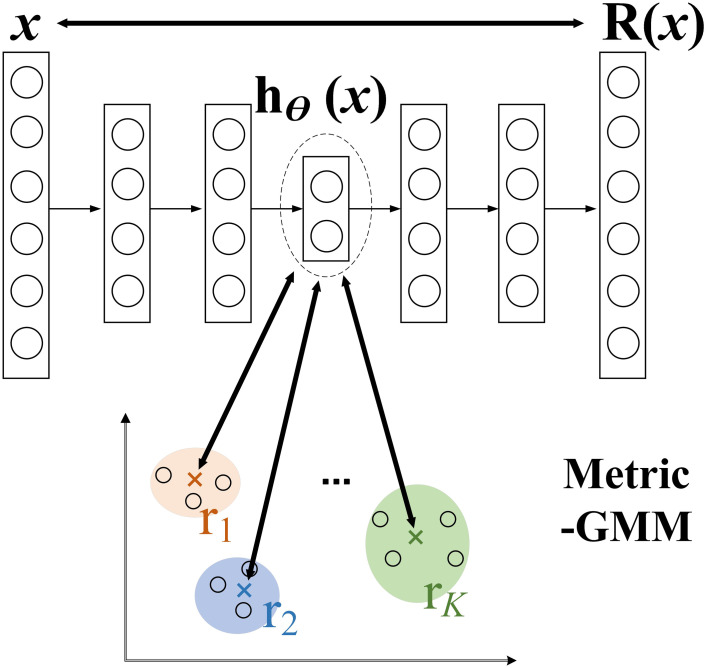
The clustering algorithm for time series data proposed in this article.

This paper designs a novel clustering algorithm for time series data, the framework of which is shown in Fig 2. The algorithm consists of two main parts.

- VAE based on Gated Recurrent Neural Network (GRU). VAE can reduce the number of dimensions in the data set to avoid complex distance calculations. The VAE design based on GRU can effectively retain the timing information of different time series.
- Aggregation level clustering. The algorithm takes the potential spatial distributions given by VAE and inputs these distributions into Gaussian distributions in a hierarchical manner, merging the two Gaussian distributions most likely to merge.

### 3.1 Variational autoencoder based on metric learning

The function of the variational autoencoder is to extract the potential spatial distribution of the input data and provide this potential distribution to the agglomerative hierarchical clustering algorithm, which can avoid the complex distance calculation problem faced by the traditional clustering algorithm, and the variational autoencoder built based on the GRU can effectively consider the time attribute of the original data. As shown in Fig 1, the input *x* goes into the input layer, and the weights (i.e., arrows) are then trained to make the output as close as possible to the input. If the input and output data are highly similar, the underlying distribution *z*_*x*_(*μ*,*σ*) in the middle contains the necessary information to reconstruct *x*. Since the encoder generally assumes that the input data conforms to the normal distribution and unsupervised learning occurs when encoding the data, this may lead to the inaccuracy of the potential distribution and further affect the subsequent clustering effect [29]. In order to ensure reliable clustering results, this section will be studied based on variational autoencoders.

#### 3.1.1. Variational autoencoder based on GRU

**Fig 3:**
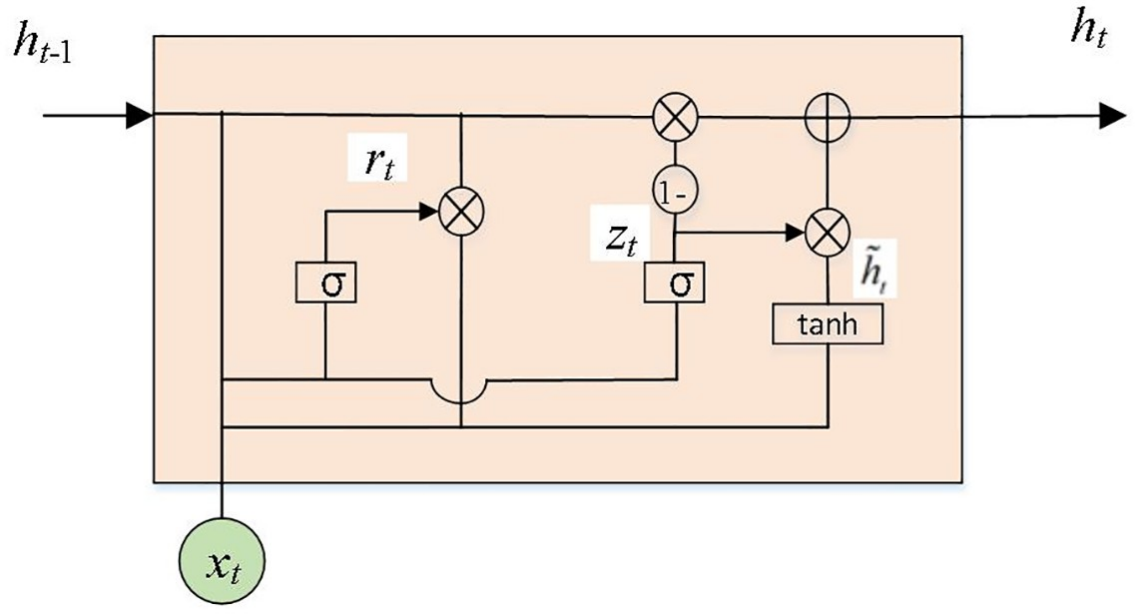
GRU structure diagram.

The basic VAE is first constructed, considering that the input data is time series data with time properties, the encoder and decoder are designed based on the GRU. GRU is improved and optimized based on the neural network structure of long and short-term memory, which reduces the number of training parameters, has a faster convergence rate, and can effectively learn time attributes in data [18]. Its structure is shown in Fig 3.

**Fig 4:**
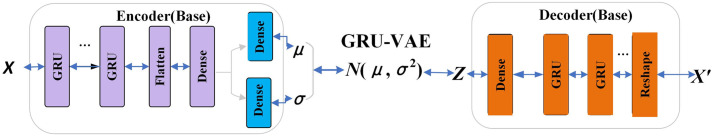
Schematic diagram of GRU-VAE structure.

The GRU consists of an update gate and a reset gate that filters historical data. Update gate (*z*_*t*_) determines the degree to which the status information of the value to be activated at the previous moment is retained to the current state. The larger the value, the more information is retained and the greater the influence of the value to be activated on the output. Reset gate (*r*_*t*_) determines the degree to which the active value of the current state is combined with the previous information. In Fig 3, the direction pointed by the arrow is the transmission direction of the data. Is the number multiplication of the matrix, 1- indicates that the data propagated forward by the link is 1-*z*_*t*_. Fig 4 shows the schematic diagram of the GRU-VAE structure.

#### 3.1.2. Loss function

After the completion of the construction of the GRU based variational autoencoder network, the optimization of the loss function of the network is considered. The loss function is the key of variational autoencoder, and the accuracy of potential space can be ensured by studying the loss function. The proposed loss function is the mean square error of the output plus the KL divergence of the prior distribution and the potential space. In particular, when the potential representation fits a Gaussian distribution, the loss function is as shown in Eq (5), where the first term is abbreviated as *L*_*recon*_ and the second term is abbreviated as *L*_*KL*_.

$$L\text{oss}=\sum_{i=1}^{n}{\left(x_{i}-g\left(f(x_{i})\right)\right)}^{2}+\sum_{i=1}^{n}\sum_{j=1}^{d}\frac{z_{i}^{2}+e^{\gamma_{i}j}-1-\gamma_{ij}}{2}$$ (5)

Where *x*_*i*_ is the input, g(*f*(*x*_*i*_)) is the reconstruction of *x*_*i*_, *n* is the number of data points, *d* is the dimension of the potential space, *z*_*i*_ is the potential representation of *x*_*i*_, *γ*_*i*,*j*_ is a transformation of the standard deviation of the variational middle layer.


$$\gamma_{i,j}=\text{log}(\sigma_{i,j}^{2})$$ (6)

Current variational autoencoders have problems of over-dependence on KL divergence and pure unsupervised learning of input data when facing time series data input, which may make the learned potential representation too simple or distorted, and it is difficult to capture the complex structure of the data adequately, thus affecting the clustering effect. Therefore, in this section, the loss function of variational autoencoder is optimized to adapt to time series data.

A:KL divergence improvement

The KL divergence term imposes some limitations on the prior distribution of the potential representation, but the original KL divergence may not be enough to improve the clustering quality in some cases, and excessive KL divergence regularization may also make the model more conservative and inhibit exploration of a wider region in the potential representation space, resulting in the potential representation being unable to effectively characterize the original data distribution. Therefore, by improving the KL divergence, we can better guide the learning of potential representations and thus improve the clustering effect. Firstly, we improve the KL divergence. The improvement is shown in Eq (7).

$$L\text{oss}=\sum_{i=1}^{n}{\left(x_{i}-g\left(f(x_{i})\right)\right)}^{2}+\sum_{i=1}^{n}\sum_{j=1}^{d}\frac{\lambda z_{i,j}^{2}+e^{\gamma_{i,j}}-1-\nu \gamma_{i,j}}{2}$$ (7)

Where the hyperparameter *λ* is the weight coefficient of the term *z*^2^, minimizing *z*^2^ is equivalent to penalizing *z* away from the origin, meaning that the potential space representation *z*_*x*_ is encouraged to remain in a finite space with a soft boundary, with high *λ* limiting the finite space, while low *λ* allows *z*_*x*_ to be distributed over a larger space. The hyperparameter *υ* is connected to *γ*, which is the logarithm of the variance used by the variable stratification, and when the variance is small, that is, the point moves very little in the potential space, *γ* takes a very negative value. Thus, large *υ* penalizes small variances and forces *z*_*x*_ to cover a larger area.The parameter *λ* is set to 0.05 by default and *υ* is set to 3 by default according to the experimental test results.

Based on Eq (7), the model can be guided to learn more representational and generalized potential representations. This improvement makes the training more stable, balances the learning of potential representations, and improves the diversity of generated samples, making the model more adaptable to the distribution of different input data.

B: Measure the loss item

**Fig 5:**
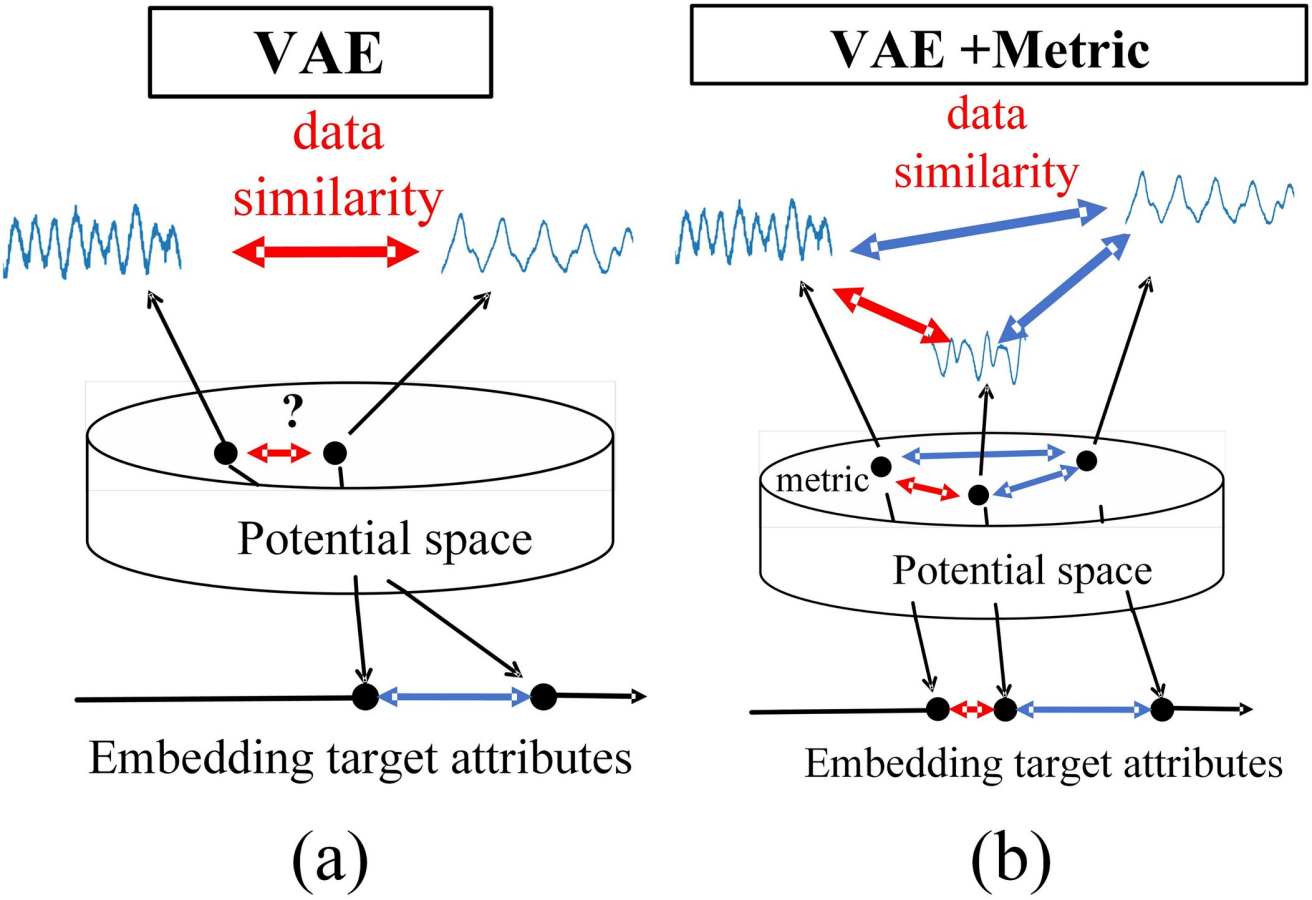
Schematic diagram of VAE based on metric learning.

In traditional VAE, the encoder maps the input data to a low-dimensional representation in a potential space in a purely unsupervised manner [16]. This low-dimensional representation is often treated as a hidden variable distribution and is often used to generate a reconstruction of the original data. However, the representation of this hidden variable distribution may not be optimal, it does not use label information about the input data, as shown in Fig 5(a), and there is a lack of effective measurement between the different distributions of the potential space, which will hinder the implementation of the clustering algorithm.

This section will use the idea of tag-based guidance to build discriminative VAE potential spaces that are more suitable for data enhancement. The key is to introduce supervisory information into the model to improve the representation of potential spaces, thereby improving the capture of data structures and effective measurement of category information in potential spaces. These improvements help to better perform clustering tasks in potential Spaces. Therefore, a metric learning loss function is introduced in this section to facilitate encoders to generate more discriminative feature representations. As shown in Fig 5(b).

**Fig 6:**
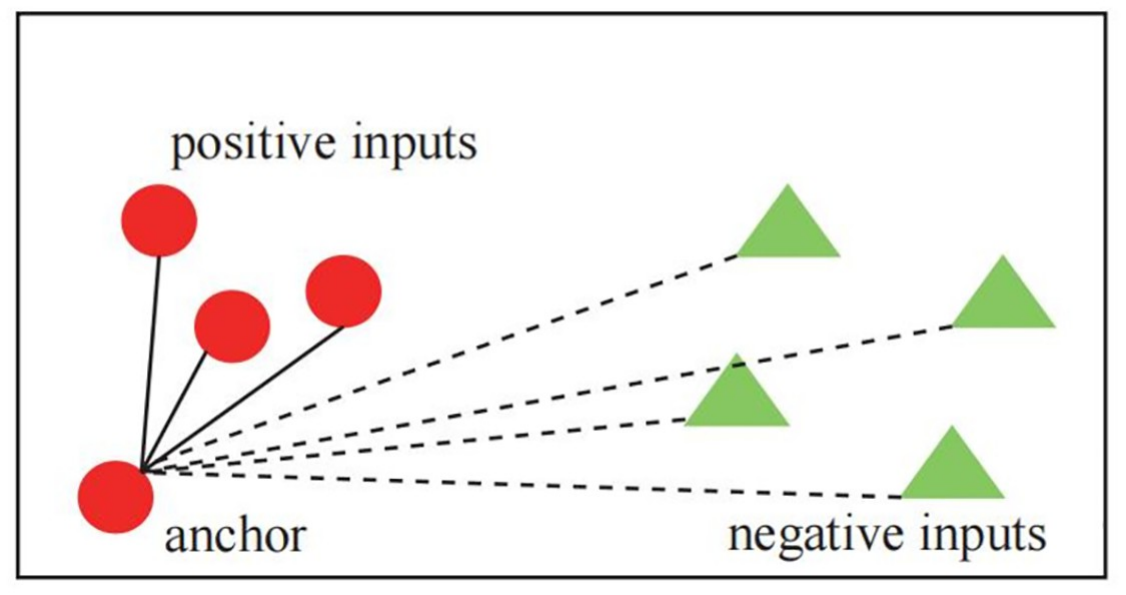
Triplet loss diagram.

In this section, triplet loss is selected as the specific implementation of metric learning, and triplet loss is shown in Fig 6.

Triplet loss *L*_*triplet*_(·), often encountered in classification Settings, is used to measure the distance between input triples. First define that *L*_*triplet*_(·), anchor/basic inputs (for example, an image of a dog) *x* (*b*), positive inputs (for example, a rotating image of a dog) *x* (*p*), and negative inputs (for example, an image of a cat) *x* (*n*) are required. The purpose of constructing this loss is to minimize the distance between the anchor point and the positive point while maximizing the distance between the anchor point and the negative point [29]. More precisely, given the separation margin *ρ*, the triples are encoded as *z* (*b*), *z* (*p*), and *z* (*n*), resulting in a triplet loss function as shown in Eq (3):

$$L_{\text{triplet}}(\text{.})=\text{max}\left\{0,{\left\|z^{\left(\text{b}\right)}-z^{\left(\text{p}\right)}\right\|}_{q}+\rho -{\left\|z^{\left(\text{b}\right)}-z^{\left(\text{n}\right)}\right\|}_{q}\right\}$$ (8)

Thus, minimizing Eq (8) results in a structured space where the positive and negative pairs are clustered together and separated by the interval *ρ*.

Metric learning is usually combined by including an additional metric loss term in the VAE loss function to achieve a discriminative VAE potential space based on metric learning. That is, for the basic input x ^(b)^, create a relative set of positive points D_p_(*x* ^(b)^; *η*) = < *x* ∈ D: | *f* (*x* ^(b)^) − *f* (*x*) | < *η* > and negative points of relative collection of D_p_ (x ^(b)^; η) = < *x* ∈ D: |f(*x* ^(b)^) − f(*x*)|≥ *η* >, where *η* is the threshold for determining the distance, as determined by the relevant properties and labels of the data, this can be preliminarily measured based on the Manhattan distance. At this stage, *L*_*triplet*_(·) can be applied during the training phase of VAE to introduce useful metrics in *Z*.

The triplet loss function *L*_*triplet*_ can be introduced into the loss function of VAE to obtain a new loss function as shown in Eq (9):

$$L_{\text{VAE}-\text{triplet}}=L_{\text{recon}}+\alpha L_{\text{KL}}+\beta L_{\text{triplet}}$$ (9)

*α* and *β* are the weight coefficients of KL loss term and improved triplet loss term respectively, which are used to balance the three loss functions.

The introduction of metric-based learning satisfies the following three clustering attributes. Property 1: Two points close to each other in the potential space should be close to each other in the input space. Property 2: Two points close to each other in the input space should be close to each other in the potential space; Property 3: Based on the underlying representation, the output obtained by the decoder should be as similar as possible to the input.

#### 3.1.3. Suitable for output of cohesive hierarchical clustering

The variational autoencoder designed in this paper generates four different outputs and passes them to the coacervation layer as follows:

Reconstructed input: It is important to check that the rebuild is similar to the input. The properties 1 and 2 discussed above are only valid if the reconstruction is good, that is, when property 3 is satisfied, because similar and dissimilar objects are measured according to how similar or dissimilar they are reconstructed, rather than the actual input.

Potential expressed expectation: E[*z*]. E[*z*] is the most accurate reconstruction of the underlying representation of the input. Therefore, these are the potential representations that will be used for clustering. Ideally, clusters should be formed that are easy to separate.

Covariance matrix of *z*: Cov[*z*]. The covariance matrix of *z* (defined by *γ*_*i*,*j*_) can be used to limit the size of the cluster.

Sample of potential Spaces:. Here, *Ɛ* is standard Gaussian noise. This output is used to check the amount of space used. Ideally, this output should spread the points throughout the space, indicating that the space is continuous in some way.

Four outputs are used to verify that the final results are reliable and provide data to the clustering algorithm.

### 3.2. Agglomerative hierarchical gaussian mixture model

After the input data is processed in Section 3.1, an accurate potential distribution *z*_*x*_ can be obtained. This section will implement a clustering algorithm based on potential distribution *z*_*x*_. Unlike clustering algorithms based on the distance between two clusters, the standard deviation provided by VAE will be used to fit the Gaussian distribution on the clusters and merge the clusters leading to the maximum log-likelihood, that is, in the process of merging clusters, the log-likelihood degree is calculated for each possible merging scenario and the cluster with the maximum log-likelihood value is selected for merging. This gives the user information about the number of possible clusters in the data, while allowing for the formation of more complex cluster geometers.

#### 3.2.1. Clustering standard

Merge based on the likelihood of merger, that is, choose the merger that results in the least reduction in the total likelihood. If clusters *i* and *j* are merged, let *l*_*k*_ represent the log-likelihood sum of the cluster at the *k*_*th*_ iteration, and let *l*_*k*+1_ represent the log-likelihood sum of the cluster at the *k*+1 iteration. The merge criteria can then be expressed as a distance minimization problem with the following representation.


$$d_{k}(i,j)=l_{k}-l_{k+1}(i,j)$$ (10)

More complex models will always have higher likelihood values because they fit the data better. However, when the model is constrained, such as merging two clusters, the total likelihood value decreases. Therefore, the goal is generally to select the combination that results in the smallest total likelihood decline in order to obtain the maximum possible likelihood under constraints. In doing so, a hierarchy of models is formed, with each model being the most likely model under some constraints. In terms of distance, the merging criteria are:

$$\begin{array}{c} \left(i_{\left[k,merge\right]},j_{\left[k,merge\right]})={\text{argmin}}_{i,j}d_{k}(i,j\right)\\ l_{k}=l_{k}\left(\left(i_{\left[k,merge\right]},j_{\left[k,merge\right]}\right)\right) \end{array}$$ (11)

However, in clustering, it is usually preferred to connect small clusters first rather than large clusters. This is because there may be special outliers in the data that belong to separate clusters and should not be merged with other points or clusters. Although such a merger is less likely, its overall impact is relatively small due to the small number of outliers. In contrast, merging large clusters may involve hundreds of points, and even if the impact of each point is small, the overall impact will be significant. To solve this problem, the average reduction of the sum of logarithmic likelihood is used as the merge criterion to better balance the effect of the merger.

$$d_{k}(i,j)=\frac{l_{k}-l_{k+1}(i,j)}{\left(n_{i}+n_{j}\right)}$$ (12)

*n*_*i*_ and *n*_*j*_ are the number of objects in cluster *i* and *j* respectively.

#### 3.2.2. Stop criterion

Ideally, the logarithmic likelihood of the data should be a monotone function of the number of data points. Simply choosing the number of clusters that maximize log-likelihood often results in each point being placed in its own cluster, a kind of overfitting by definition that makes clustering analysis redundant. The cohesive hierarchy algorithm creates a hierarchy that continuously merges clusters until all data points are in the same cluster.

However, sometimes too many merges can cause the model to underfit the data, and the log-likelihood value becomes very low, rendering the cluster analysis meaningless. One intuitive criterion is to stop when the merge becomes less natural, such as having an average value far away from the point, a large covariance matrix on the ellipsoid or an odd shape. This condition can be detected by a log-likelihood graph measurement, which is indicated by a large drop.

When deciding to stop merging, it is difficult to use standardized tests to determine the number of parameters in the model because the number of data points merged in many steps of the hierarchy is relatively small. Although these obstacles are difficult to overcome, plausibility based models can help detect unnatural clusters by studying probability graphs. In this paper, the total log-likelihood and visual representation will be presented, and clustering can be stopped when the total log-likelihood is greater than 0. In addition, estimating the Gaussian distribution and calculating the likelihood value consumes more computing power than calculating the distance. This difference makes GMM methods infeasible even for relatively small data sets. To solve this problem, a normal coacervation algorithm with average links is integrated into the algorithm to improve the clustering speed. In the early stages when the clusters are small and abundant, the GMM method will give the same result as the distance method if the initialization is reasonable. When the clusters are larger and initialization is less important, the likelihood merge criteria are used for merging.

## 4. Experimental analysis

Experimental conditions: computer configuration.

Intel (R) Core (TM) i5-7500 CPU @ 3.40GHZ

Before proceeding with experimental validation, let us first detail the parameter design of the proposed time series data clustering algorithm. The VAE utilizes a three-layer architecture in both the encoder and decoder, specifically incorporating GRUs to capture the dynamic properties of time series. The encoder is composed of layers with 128, 64, and 32 units respectively, while the decoder features a symmetric structure to ensure the coherence of data reconstruction. The latent space dimension has been set to 32 to balance representational capability and computational efficiency. The weights of the KL divergence loss term (*α*) and the triplet loss weight (*β*) are initialized at 1 each to foster balanced model training. The metric learning component employs a fixed sampling strategy and triplet loss based on Euclidean distance, aiming to improve the representational accuracy of the latent space. For hierarchical clustering, the average linkage criterion and predetermined merging standards are adopted, coupled with the proposed stopping criteria, thus reducing computational costs and enhancing the interpretability of clustering outcomes. This comprehensive parameter setup reflects a thorough consideration of the characteristics of time series and the quality of clustering, aiming to enhance the clustering performance of time series data through the integration of gated neural networks and metric learning.

This paper uses two standard measures to evaluate clustering performance, including clustering accuracy (ACC) and normalized Mutual Information (NMI). ACC finds the best mapping between true and predictive clustering labels. NMI finds a standardized measure of similarity between two different labels of the same data point. The calculation equations are as follows:

$$ACC=max_{m}\frac{\sum_{i=1}^{N}1\{l_{i}=map(cl_{i})\}}{N}$$ (13)

$$NMI=\frac{I(l;cl)}{\text{max}(H(l),H(cl))}$$ (14)

Where *l*_*i*_ and *cl*_*i*_ represent the real label and predicted label of data point *x*_*i*_, respectively. Map(.) represents the best mapping between the predicted label and the real label of the data point. I = (*l*; *cl*) represents the true label of all data points *l* = {*l*_1_,*l*_2_,…, *l*_*N*_] and predictive clustering assignment *Cl* = {*cl*_1_,*cl*_2_,…, *cl*_*N*_] mutual information between. H(.) represents the entropy function. Both ACC and NMI ranges are within the interval [0, 1], and the higher the score, the higher the clustering performance [20].

### 4.1. Encoder potential spatial accuracy verification

First, the accuracy of the potential space encoded by the encoder in the variational autoencoder based on metric learning is tested. The potential space obtained by the encoder will be used as a distribution for sampling output by the decoder. If the output obtained by the decoder is similar to the original input data, it can be proved that the potential representation *z*_*x*_ contains the necessary information needed to reconstruct *x*. That is, the potential space meets the requirement of clustering accuracy.

Therefore, the potential spatial accuracy is judged in this section by the similarity between the decoder output and the original data. Three evaluation criteria in TimeGAN [30] are used: (1) Diversity—the generated data distribution should cover the real data distribution, that is, t-SNE analysis is applied to the original data set and the generated data set, which visualizes the degree of similarity between the generated sample distribution and the original sample distribution in two-dimensional space, and gives a qualitative assessment of diversity. (2) Fidelity (discrimination score)—The generated data should be no different from the real data. To measure similarity quantitatively, a post-hoc time series classification model is trained (by optimizing the 2-layer LSTM) to distinguish between the original data set and the sequence of the generated data set, which gives a quantitative assessment of fidelity. (3) Usefulness (prediction score)—when used for the same prediction purpose, the generated data should be as useful as the real data, that is, the generated data should inherit the prediction characteristics of the original data, using the "synthetic Training and Real testing" experiment, that is, using the synthetic data to predict the next value based on the LSTM with a two-layer GRU. Using mean square error to quantify usefulness, the mean square error equation is shown in Eq (15).


$$MSE=\frac{1}{n}\sum_{i=1}^{n}{\left(y_{i}-{\hat{y}}_{i}\right)}^{2}$$ (15)

**Fig 7:**
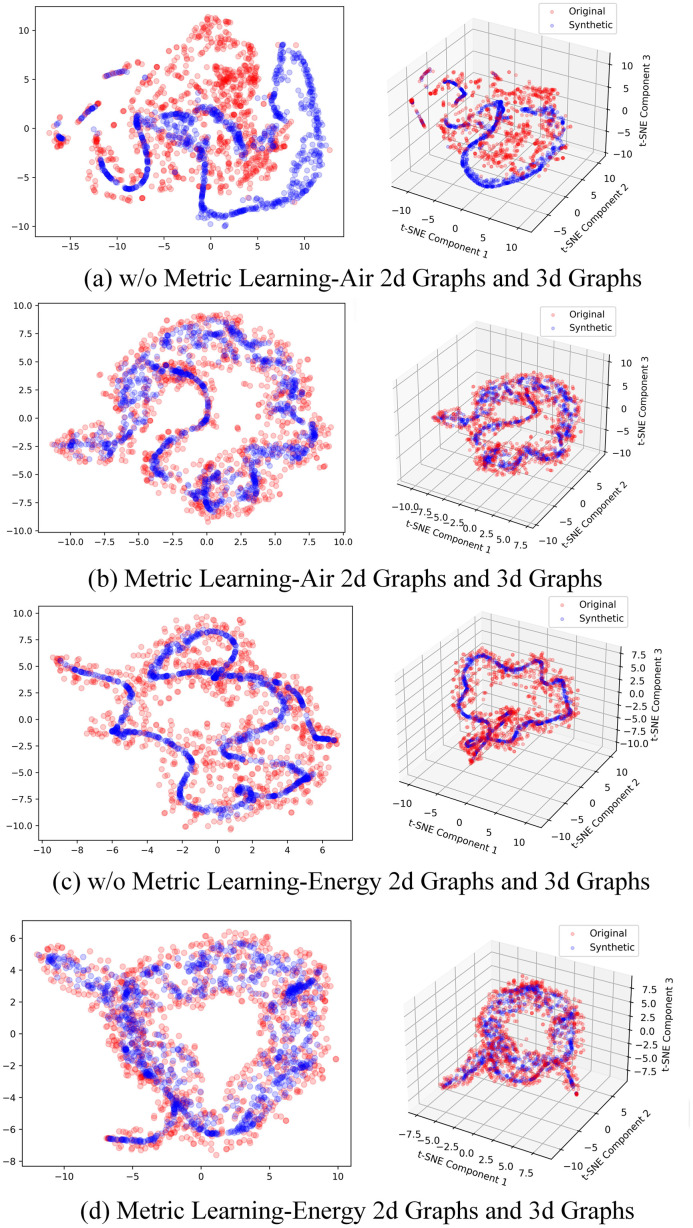
Verification of potential spatial accuracy.

Air and energy data sets in UCI data sets were selected to verify the potential spatial accuracy. The sample number of air data set was 9333, and the feature number was 15, and the sample number of energy data set was 19711, and the feature number was 28 [31]. Through the specific analysis of the above three evaluation criteria, Fig 7 and Table 1 are obtained as follows.

**Table 1:** Quantitative verification of potential spatial accuracy.

Evaluation Criterion	Methods	Air	Energy
**Discrimination Score**	**Proposed Algorithm**	**0.176±0.010**	**0.129±0.003**
	**w/o Metric Learning**	**0.430±0.050**	**0.306±0.001**
**Prediction Score**	**w/o Metric Learning**	**0.399±0.020**	**0.281±0.002**
	**Proposed Algorithm**	**0.187±0.005**	**0.155±0.002**
	**Original data**	**0.180±0.001**	**0.153±0.002**

A diversity-based qualitative assessment was conducted, focusing on air quality and energy data. The results in Fig 7(a) reveal that, without metric learning, the generated data did not completely encompass the original data; this was particularly evident in three-dimensional space, where the similarity between generated and original data significantly diminished. This demonstrates that, on the air quality dataset, the absence of metric learning compromised the latent space representation’s accuracy obtained by the encoder, potentially affecting clustering accuracy. Conversely, as seen in Fig 7(b), the introduction of metric learning enabled a more uniform coverage of the original data, with the distributions appearing closer in three-dimensional space. This indicates that metric learning effectively enhances the encoder’s latent space accuracy, facilitating time series data clustering. For the energy data, Fig 7(c) illustrates that without metric learning, there was a lack of uniformity in data coverage. The three-dimensional view more clearly showed that, without metric learning, the algorithm primarily learned the general trend of the original data, overlooking specific details, which could impact the accuracy of subsequent clustering. In contrast, as shown in Fig 7(d), the inclusion of metric learning not only ensured uniform coverage of the original data but also exhibited greater detail similarity in the three-dimensional view, affirming that metric learning secures the accuracy of the encoder’s latent space.

By comparing air quality and energy data outcomes with and without metric learning, it is evident that metric learning is essential for the encoder’s ability to learn the latent distribution space during the encoding process of the original data, offering essential support for the clustering operations.

Table 1 displays the experimental results validating latent space accuracy, featuring discriminative and predictive scores across different methods for the Air and Energy datasets. Discriminative and predictive scores measure differences between generated and original data, where smaller values signify higher fidelity and utility.

The proposed algorithm’s performance notably exceeds that of other methods on both datasets. Specifically, for the Air dataset, methods without metric learning show a discriminative score of 0.430±0.050, in contrast to the proposed algorithm’s score of 0.176±0.010. This demonstrates the proposed algorithm’s substantial advantage in minimizing the difference between generated and original data. Similarly, for the Energy dataset, the proposed algorithm achieves a discriminative score of 0.129±0.003, significantly lower than the 0.306±0.001 of methods without metric learning, validating the proposed algorithm’s efficiency in generating high-quality data.

For predictive scores, which assess the effectiveness of predictions made using generated data, the original data’s scores serve as benchmarks: 0.180±0.001 for Air and 0.153±0.002 for Energy. The proposed algorithm achieves predictive scores of 0.187±0.005 for Air and 0.155±0.002 for Energy, showing minimal deviations and indicating that algorithm-generated data retains the original data’s predictive qualities.

In conclusion, these quantitative results indicate that the proposed algorithm effectively minimizes the difference between generated and original data while preserving data fidelity, ensuring the accuracy of the learned latent space—a key factor for achieving meaningful and reliable clustering outcomes.

### 4.2. Cluster validation for cell load

A dataset comprising electricity demand time series data from a residential area, as measured by a power company’s Metering Center, containing 1096 samples spanning 24 hours and featuring two types, was utilized to validate the clustering algorithm detailed in this study. The weights for the KL divergence loss term (*α*) and the triplet loss (*β*) were finalized following a series of experiments and assessments. Initially, parameter adjustments started from *α* = 1 and *β* = 1, guided by the data characteristics and preliminary experimental outcomes. Using the Bayesian optimization algorithm, iterative testing was conducted to observe the model’s clustering performance and time series reconstruction accuracy under diverse parameters.

Throughout the experiment, it was observed that incrementally increasing *α* to 1.5 enhanced the model’s ability to reconstruct time series and capture the data’s intrinsic periodicity, while further increases in α offered minimal improvements and could lead to excessive regularization constraints. Adjusting *β* to 2 enhanced differentiation of data among categories in the latent space, with higher *β* values not significantly improving cluster separation. After a comprehensive consideration of reconstruction quality, category separation effects, and model generalization capabilities, the weights for the KL divergence loss term (α) were ultimately set at 1.5, and the triplet loss (*β*) at 2. This parameter configuration not only guarantees effective category separation in the latent space but also optimizes clustering performance, ensuring that time series data characteristics are effectively captured. Based on this setting, we obtained compelling experimental outcomes.

**Fig 8:**
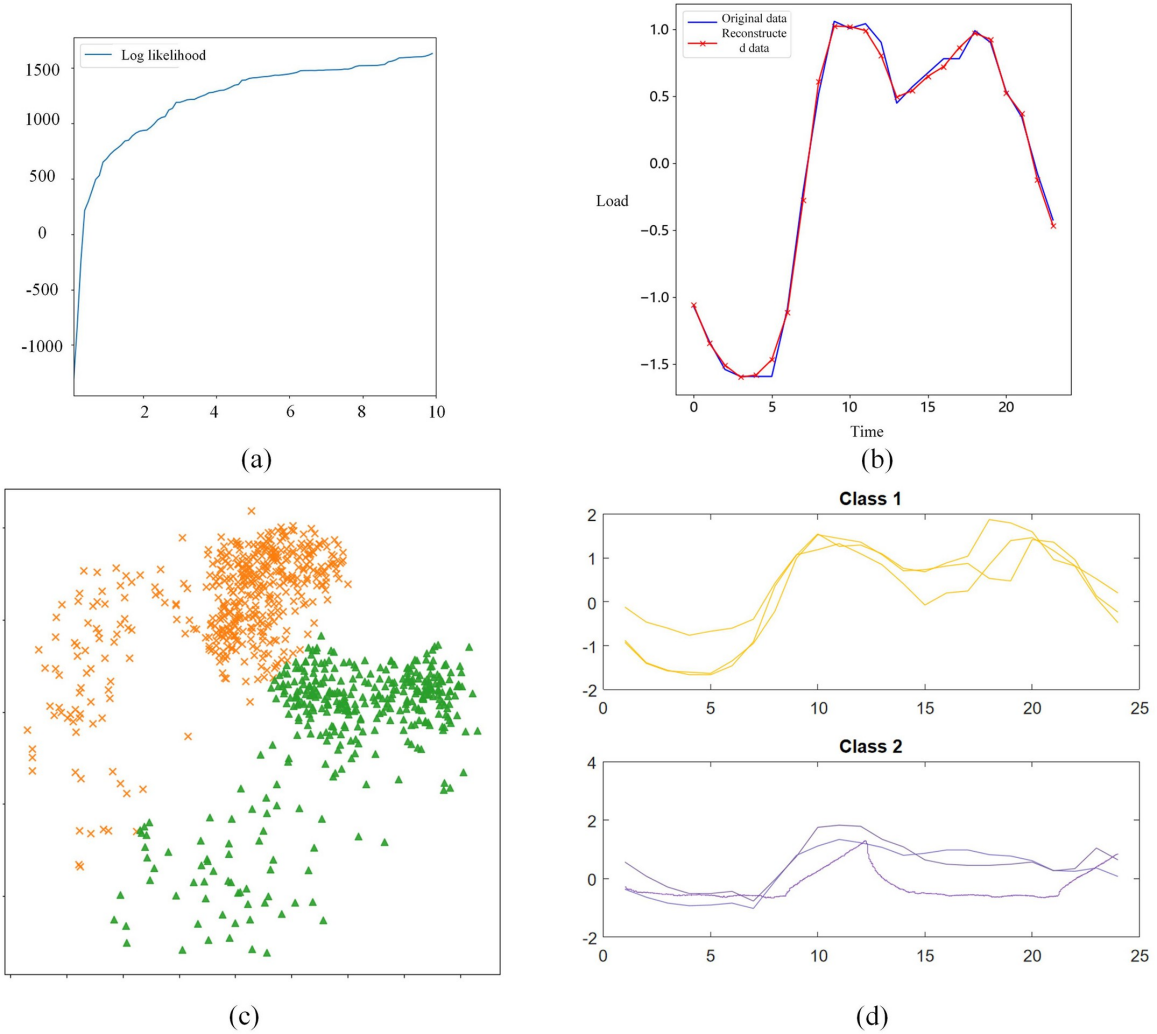
Electricity demand clustering test.

A qualitative analysis was initially conducted. Fig 8(a) demonstrates that with the number of clusters k set to 2, this configuration already satisfies the merging criteria based on merging possibilities. Given the speed requirements, this study opts for k = 2 in sample clustering. Clustering-based data can precisely reconstruct the original data curve, ensuring satisfaction of properties 1 and 2. Cluster diagram 8(c) showcases the clustering effect achieved by this study’s algorithm. The advantage of soft clustering offered by this study’s algorithm is evident, enabling relatively precise sample clustering. Specifically, a notable separation is observed on dimension 1 (horizontal axis), particularly between the upper boundary of the orange cluster and the lower boundary of the green cluster, illustrating the algorithm’s effectiveness in distinguishing different data clusters on this dimension. Meanwhile, despite slight overlaps in some areas of dimension 2 (vertical axis), good separation is nevertheless maintained. Fig 8(d) presents the typical sample curves for each clustered sample class, revealing clear electricity usage trends that facilitate further modeling and analysis of user behavior.

Table 2 offers a comparative analysis of latent space accuracy validation across various clustering algorithms, including K-Means [7], GP-DBSCAN [32], FC-Kmeans [14], EM-VAE [28], and the algorithm proposed in this paper. Performance evaluation for these algorithms was based on four key metrics: accuracy (ACC), normalized mutual information (NMI), runtime (in seconds), and the determined number of clusters.

**Table 2:** Comparison of relevant algorithm indicators.

Methods	ACC	NMI	Speeds	Clusters
**Means**	**0.579**	**0.523**	**63.1s**	**4**
**GP-DBSCAN**	**0.713**	**0.657**	**79.3s**	**3**
**FC-Kmeans**	**0.795**	**0.712**	**73.6s**	**3**
**EM-VAE**	**0.721**	**0.669**	**55.3s**	**3**
**Proposed Algorithm**	**0.862**	**0.858**	**27.6s**	**2**

Results demonstrate that the algorithm proposed in this paper outperforms the comparative algorithms across all metrics. Specifically, the proposed algorithm exhibits exceptional performance on the ACC metric, reaching 0.862, in contrast to the traditional K-Means algorithm, which achieves only 0.579. This indicates a roughly 48.9% higher accuracy over K-Means. Similarly, against GP-DBSCAN, FC-Kmeans, and EM-VAE, the ACC improvements are 20.9%, 8.4%, and 19.6%, respectively, highlighting the proposed algorithm’s marked efficiency in discerning the data’s inherent structure.

In the realm of NMI, the proposed algorithm also shows superior performance, achieving 0.858, which surpasses K-Means, GP-DBSCAN, FC-Kmeans, and EM-VAE by 64.1%, 30.6%, 20.5%, and 28.3%, respectively. This further illustrates the proposed algorithm’s advantage in maintaining the data’s true structure, effectively capturing the dataset’s real relationships during the clustering process.

Speed is a crucial metric in the evaluation of clustering algorithms. Regarding speed, the algorithm proposed in this study markedly surpasses the four other algorithms, completing computations in just 27.6 seconds. It achieves speeds 56.3% faster than K-Means, 65.2% over GP-DBSCAN, 62.5% beyond FC-Kmeans, and 50.1% quicker than EM-VAE. These outcomes demonstrate the proposed algorithm’s high accuracy and efficiency, making it well-suited for large datasets and scenarios that demand quick responses.

Accurately identifying the number of clusters is another critical evaluation criterion for clustering algorithms. The proposed algorithm precisely determined the true number of clusters to be 2, while other algorithms either overestimated the cluster count or underperformed. This indicates the proposed algorithm’s superior accuracy and adaptability in analyzing data structures.

In conclusion, the proposed algorithm excels across all evaluated metrics, notably in the accurate determination of cluster numbers, precision, and speed.

### 4.3. Industrial load cluster verification

**Fig 9:**
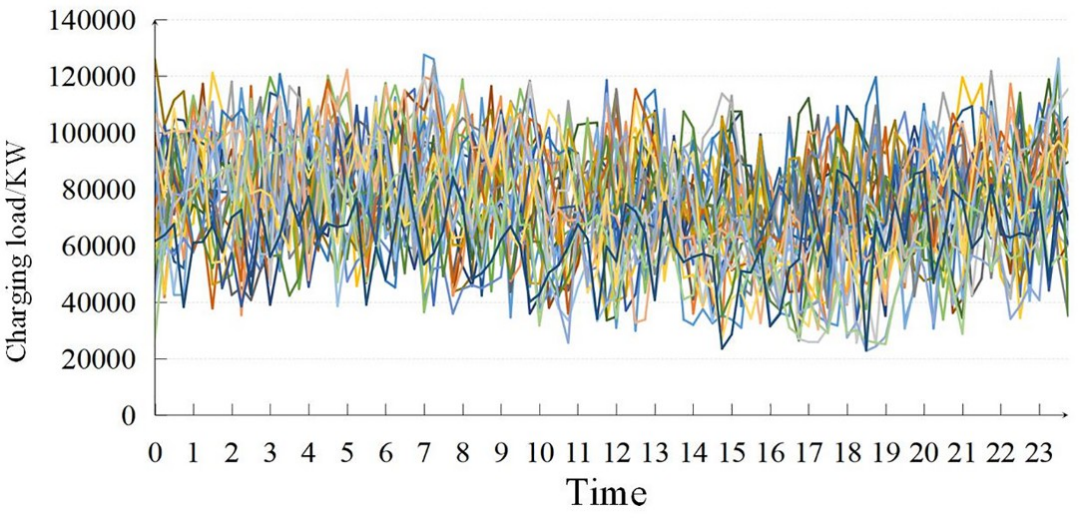
Large industrial load data curve.

Clustering validation utilized electricity load data from a large industrial factory in a specific region. Operating continuously, the factory’s data spans 24 hours, with collections every 15 minutes, yielding 96 points across 7 categories. As Fig 9 illustrates, the load data curve shows significant randomness and fluctuations, attributed to the factory’s diverse load equipment types and their spiky, fluctuating load characteristics. Following the strategy outlined in Section 4.2, the initial KL divergence loss weight (*α*) was set to 1.5 to capture the data’s volatility and inherent structure. Additionally, the triplet loss weight (*β*) was established at 2.5 to improve differentiation among load types, accommodating the subtle variations and complexity of the factory’s load characteristics.

The clustering algorithm proposed in this paper is used for cluster analysis. Different from the previous examples, this example belongs to clusters with more clustering centers, so as to verify the effectiveness of the proposed algorithm for clustering.

**Fig 10:**
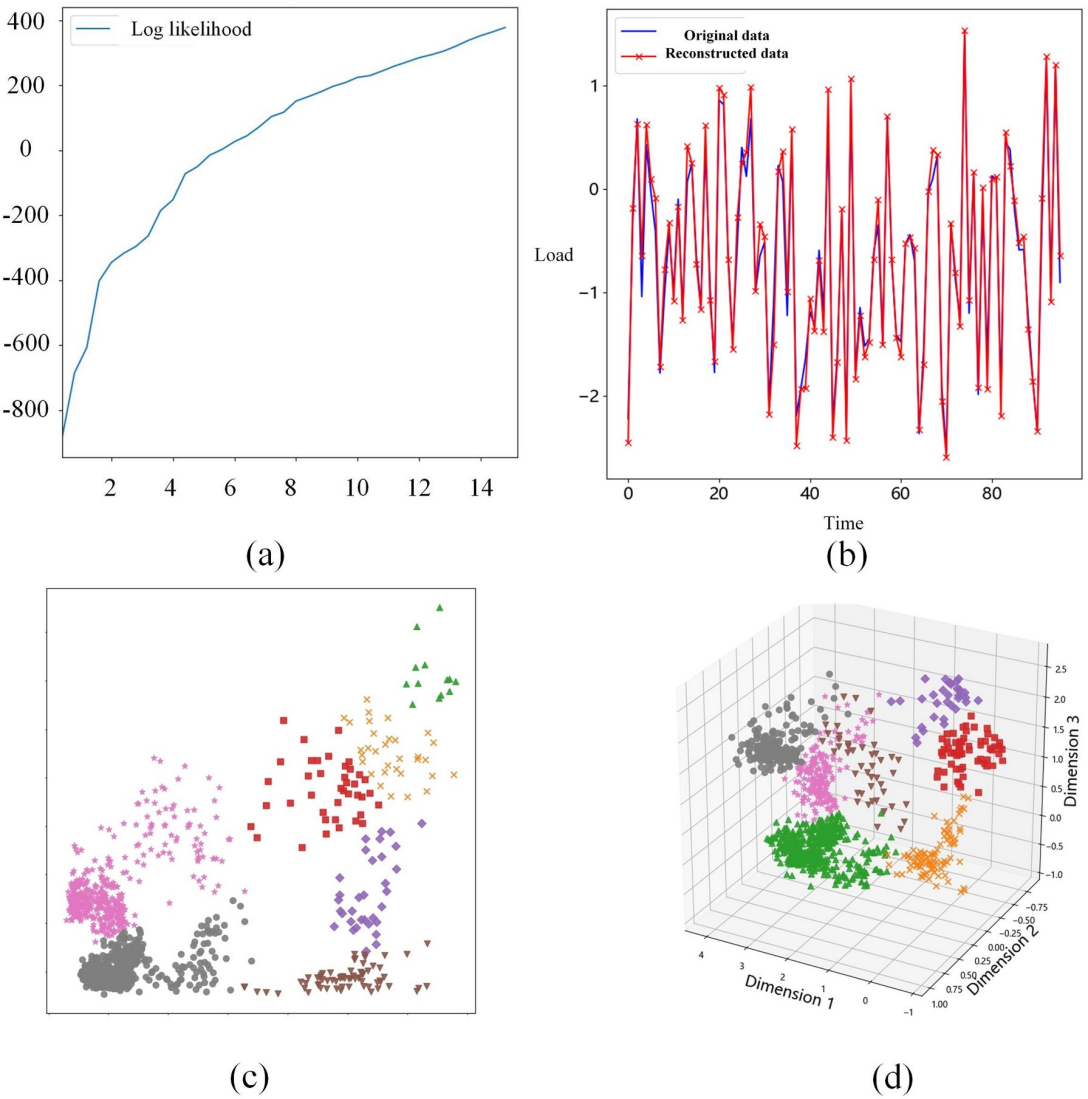
Cluster testing of large industrial load data.

Fig 10(a) clearly shows that selecting a cluster number k as 7 meets the merging criteria; therefore, k = 7 was chosen. Fig 10(b) demonstrates a high coincidence between the original and reconstructed load data curves, indirectly verifying the accuracy between the VAE’s encoder and decoder, and confirms accurate partitioning in the latent space *z*_*x*_. Upon confirming the cluster count, the algorithm’s clustering is depicted in Fig 10(c) and 10(d). A comprehensive analysis of the two-dimensional results in Fig 10(c) and three-dimensional results in Fig 10(d) reveals the algorithm’s effectiveness in identifying and distinguishing diverse patterns and structures across data spaces of varying dimensions. This suggests that in both reduced two-dimensional and original or higher-dimensional three-dimensional spaces, the proposed algorithm accurately partitions the data’s intrinsic groups, demonstrating its flexibility and robustness.

The two-dimensional clustering analysis showcases the algorithm’s efficiency with a clear delineation between clusters and consistent membership within each cluster on reduced-dimensional data. Specifically, clusters distinguished by different markers and colors on the two-dimensional plane highlight the algorithm’s capability to precisely capture and reflect data’s varying characteristics and distributions. For instance, the stark separation between the purple diamond and orange cross clusters, along with the unique distribution patterns of the pink star and gray circle clusters, emphasizes the algorithm’s adaptability and precision across diverse data distributions.

The three-dimensional clustering analysis deepens the understanding of data structures through the spatial representation of clusters, demonstrating that the proposed algorithm excels not just in two-dimensional spaces but also in leveraging additional information to further distinguish data in high-dimensional scenarios. Spatial isolation between clusters and tight aggregation within them further validate the clustering results’ reliability and the algorithm’s effectiveness.

**Fig 11:**
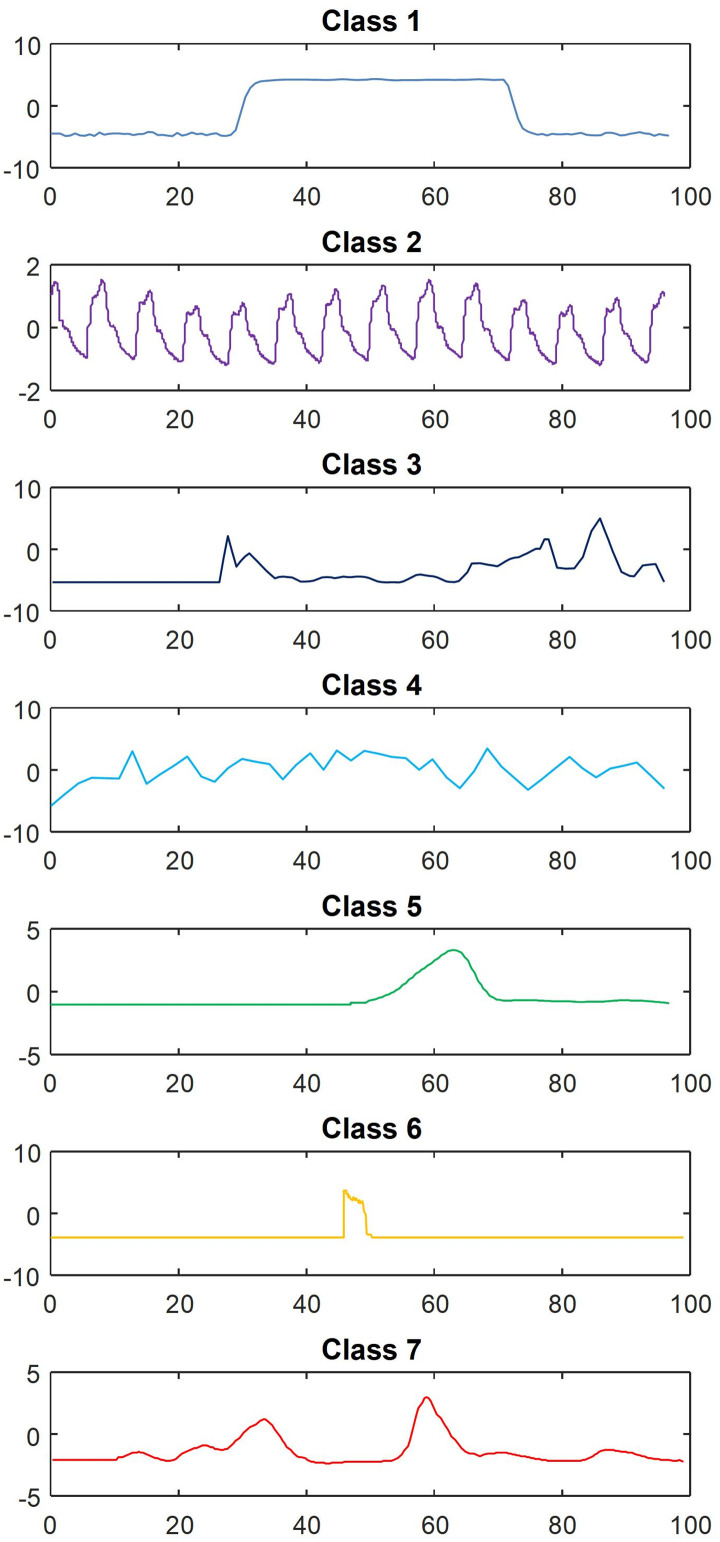
Load characteristic curve diagram.

Drawing upon the clustering results, Fig 11 illustrates the load characteristic curves of seven types, revealing significant randomness and fluctuation. This variability can be attributed to the factory’s numerous load equipment types and their spiky, fluctuating load characteristics.

Table 3 showcases a comparison of the performance of various clustering algorithms on a large industrial electricity load dataset, including K-Means, GP-DBSCAN, FC-Kmeans, EM-VAE, and the algorithm proposed in this study. Collectively, these metrics highlight the performance and suitability of clustering algorithms, particularly in processing complex industrial data.

**Table 3:** Comparison of relevant algorithm indicators.

Methods	ACC	NMI	Speeds	Clusters
**K-Means**	**0.593**	**0.546**	**72.4s**	**8**
**GP-DBSCAN**	**0.732**	**0.691**	**89.3s**	**8**
**FC-Kmeans**	**0.754**	**0.699**	**85.2s**	**9**
**EM-VAE**	**0.738**	**0.727**	**87.6s**	**8**
**Proposed Algorithm**	**0.887**	**0.895**	**43.9s**	**7**

Regarding the ACC metric, the algorithm proposed in this study significantly outperforms all others in the comparative analysis. With an ACC value of 0.887, it surpasses the basic K-Means by 49.6%, the advanced GP-DBSCAN by 21.2%, FC-Kmeans by 17.6%, and EM-VAE by 20.2%. This marked improvement underscores the proposed algorithm’s distinct advantage in accurately clustering large-scale industrial electricity load data, especially in identifying complex data structures and patterns with precision.

NMI serves as a crucial metric to measure the alignment of clustering results with the actual data distribution. Achieving 0.895 in NMI, the proposed algorithm demonstrates superiority, being 63.9% higher than K-Means, 29.5% over GP-DBSCAN, 28.0% beyond FC-Kmeans, and 23.1% above EM-VAE. This significant enhancement illustrates the algorithm’s capability in unveiling the intrinsic structure of data, notably when analyzing industrial data characterized by complex distributions.

Concerning cluster number identification, the proposed algorithm precisely identifies the presence of 7 clusters, a minor variance in comparison to other algorithms. This suggests that the proposed algorithm aligns more closely with the data’s inherent structure, offering a more accurate data segmentation, crucial for comprehending the complexity and variability of industrial data.

Regarding runtime speed, the algorithm proposed in this study exhibits exceptional efficiency, requiring only 43.9 seconds to complete the clustering process. In comparison, K-Means takes 72.4 seconds, GP-DBSCAN 89.3 seconds, FC-Kmeans 85.2 seconds, and EM-VAE 87.6 seconds. The proposed algorithm’s speed improvements are 39.4%, 50.8%, 48.5%, and 49.9% over K-Means, GP-DBSCAN, FC-Kmeans, and EM-VAE, respectively. This demonstrates that, alongside maintaining high accuracy, the proposed algorithm also offers faster clustering speeds, a critical aspect for practical applications that demand the rapid processing of large datasets, such as real-time electricity load monitoring and forecasting.

The proposed algorithm shows substantial advantages in the clustering analysis of large-scale industrial electricity load data, particularly in terms of accuracy, runtime speed, and the ability to reveal data structures. These outcomes not only confirm the effectiveness of the proposed algorithm but also underscore its superior capability in managing large-scale and complex datasets.

## 5. Conclusions

This paper presents a new clustering algorithm for time series data. When processing time series input, both encoder and decoder adopt GRU structure, which is helpful to effectively learn time information in data. In view of the current over-reliance on KL divergence and unsupervised learning when processing time series data, which may lead to oversimplification or distortion of the underlying representation learned, affecting the clustering effect, the KL divergence loss term is improved, and metric learning is introduced to ensure that the variational autoencoder can more fully capture the complex structure of the data, Improve the accuracy of potential space characterization; In addition, the sum of logarithmic likelihood is adopted as the merging criterion, and the stopping criterion is developed to realize the visualization of the number of clusters and optimize the clustering speed. Through cluster analysis on large industrial load data, the accuracy and speed of the algorithm in this paper are improved by 27.16% and 47.15% on average, which verifies the feasibility and effectiveness of the algorithm.

Clustering algorithms utilizing the Variational Autoencoder (VAE) framework struggle to adapt effectively to multivariate time series data inputs. This strategy somewhat neglects the interconnectedness among multivariate time series, often necessitating extensive preprocessing, including analysis of assumptions regarding the input data distribution. Moving forward, we aim to investigate clustering algorithms for multivariate time series data, grounded in metric learning. This strategy will thoroughly account for the influence of prior data knowledge on clustering results and will also include the validation of instances concerning the normal distribution assumption in the encoder’s latent space for time series data.

## References

[1] Zhao H, Liu J, Chen H, Chen J, Li Y, Xu J, et al. Intelligent Diagnosis Using Continuous Wavelet Transform and Gauss Convolutional Deep Belief Network. IEEE Transactions on Reliability. 2023;72: 692–702. doi: 10.1109/TR.2022.3180273

[2] Zhao H, Liu H, Jin Y, Dang X, Deng W. Feature extraction for data-driven remaining useful life prediction of rolling bearings. IEEE Transactions on Instrumentation and Measurement. 2021;70: 1–10.33776080

[3] Zhao H, Zheng J, Deng W, Song Y. Semi-supervised broad learning system based on manifold regularization and broad network. IEEE Transactions on Circuits and Systems I: Regular Papers. 2020;67: 983–994.

[4] Cheng D, Yang F, Xiang S, Liu J. Financial time series forecasting with multi-modality graph neural network. Pattern Recognition. 2022;121: 108218.

[5] Zhao H, Liu H, Xu J, Deng W. Performance prediction using high-order differential mathematical morphology gradient spectrum entropy and extreme learning machine. IEEE transactions on instrumentation and measurement. 2019;69: 4165–4172.

[6] Chu J, Liu J, Wang H, Meng H, Gong Z, Li T. Micro-Supervised Disturbance Learning: A Perspective of Representation Probability Distribution. IEEE Transactions on Pattern Analysis and Machine Intelligence. 2023;45: 7542–7558. doi: 10.1109/TPAMI.2022.3225461 36445994

[7] Holder C, Middlehurst M, Bagnall A. A review and evaluation of elastic distance functions for time series clustering. Knowl Inf Syst. 2024;66: 765–809. doi: 10.1007/s10115-023-01952-0

[8] Ikotun AM, Ezugwu AE. Boosting k-means clustering with symbiotic organisms search for automatic clustering problems. PLOS ONE. 2022;17: e0272861. doi: 10.1371/journal.pone.0272861 35951672 PMC9371361

[9] Ezugwu AE, Ikotun AM, Oyelade OO, Abualigah L, Agushaka JO, Eke CI, et al. A comprehensive survey of clustering algorithms: State-of-the-art machine learning applications, taxonomy, challenges, and future research prospects. Engineering Applications of Artificial Intelligence. 2022;110: 104743.

[10] Dilokthanakul N, Mediano PAM, Garnelo M, Lee MCH, Salimbeni H, Arulkumaran K, et al. Deep Unsupervised Clustering with Gaussian Mixture Variational Autoencoders. arXiv; 2017. http://arxiv.org/abs/1611.02648

[11] Jiang Z, Zheng Y, Tan H, Tang B, Zhou H. Variational Deep Embedding: An Unsupervised and Generative Approach to Clustering. arXiv; 2017. http://arxiv.org/abs/1611.05148

[12] Madiraju NS. Deep temporal clustering: Fully unsupervised learning of time-domain features. PhD Thesis, Arizona State University. 2018. https://search.proquest.com/openview/0445e68c9f5c08845519d369c45a94fc/1?pq-origsite=gscholar&cbl=18750

[13] Ma Q, Zheng J, Li S, Cottrell GW. Learning representations for time series clustering. Advances in neural information processing systems. 2019;32. Available: https://proceedings.neurips.cc/paper/2019/hash/1359aa933b48b754a2f54adb688bfa77-Abstract.html

[14] Caciularu A, Goldberger J. An entangled mixture of variational autoencoders approach to deep clustering. Neurocomputing. 2023;529: 182–189.

[15] Zhong Y, Huang D, Wang C-D. Deep Temporal Contrastive Clustering. Neural Process Lett. 2023;55: 7869–7885. doi: 10.1007/s11063-023-11287-0

[16] Alqahtani A, Ali M, Xie X, Jones MW. Deep Time-Series Clustering: A Review. Electronics. 2021;10: 3001. doi: 10.3390/electronics10233001

[17] Suh WH, Oh S, Ahn CW. Metaheuristic-based time series clustering for anomaly detection in manufacturing industry. Appl Intell. 2023;53: 21723–21742. doi: 10.1007/s10489-023-04594-5

[18] Li H, Liu Z, Wan X. Time series clustering based on complex network with synchronous matching states. Expert Systems with Applications. 2023;211: 118543.

[19] Oyewole GJ, Thopil GA. Data clustering: application and trends. Artif Intell Rev. 2023;56: 6439–6475. doi: 10.1007/s10462-022-10325-y 36466764 PMC9702941

[20] Afzal S, Iqbal MM, Afzal A, Bakouch HS, Aljeddani SMA. Novel Approaches to Identify Clusters Using Independent Components Analysis with Application. Mathematical Problems in Engineering. 2023;2023: e4830716. doi: 10.1155/2023/4830716

[21] Zakaria J, Mueen A, Keogh E, Young N. Accelerating the discovery of unsupervised-shapelets. Data Min Knowl Discov. 2016;30: 243–281. doi: 10.1007/s10618-015-0411-4

[22] Ruan H, Hu X, Xiao J, Zhang G. TrSAX—An improved time series symbolic representation for classification. ISA transactions. 2020;100: 387–395. doi: 10.1016/j.isatra.2019.11.018 31791613

[23] Dat DQ, Hung PD. Improvement for Time Series Clustering with the Deep Learning Approach. In: Luo Y, editor. Cooperative Design, Visualization, and Engineering. Cham: Springer International Publishing; 2021. pp. 73–83.

[24] Ienco D, Interdonato R. Deep semi-supervised clustering for multi-variate time-series. Neurocomputing. 2023;516: 36–47. doi: 10.1016/j.neucom.2022.10.033

[25] Ienco D, Interdonato R. Deep Multivariate Time Series Embedding Clustering via Attentive-Gated Autoencoder. In: Lauw HW, Wong RC-W, Ntoulas A, Lim E-P, Ng S-K, Pan SJ, editors. Advances in Knowledge Discovery and Data Mining. Cham: Springer International Publishing; 2020. pp. 318–329.

[26] Kim J, Moon N. A deep bidirectional similarity learning model using dimensional reduction for multivariate time series clustering. Multimed Tools Appl. 2021;80: 34269–34281. doi: 10.1007/s11042-020-10476-6

[27] Xu C, Chen J. Deep clustering model for time-series data based on recurrence plot and variational auto-encoder. Third International Conference on Digital Signal and Computer Communications (DSCC 2023). SPIE; 2023. pp. 97–105.

[28] Umatani R, Imai T, Kawamoto K, Kunimasa S. Time series clustering with an EM algorithm for mixtures of linear Gaussian state space models. Pattern Recognition. 2023;138: 109375.

[29] Galetzka W, Kowall B, Jusi C, Huessler E-M, Stang A. Distance-Metric Learning for Personalized Survival Analysis. Entropy. 2023;25: 1404. doi: 10.3390/e25101404 37895525 PMC10606222

[30] Seol S, Lee J, Yoon J, Kim B. Improving SOH estimation for lithium-ion batteries using TimeGAN. Machine Learning: Science and Technology. 2023;4: 045007.

[31] Arshad M, Jaskani FH, Sabri MA, Ashraf F, Farhan M, Sadiq M, et al. Hybrid machine learning techniques to detect real time human activity using UCI dataset. EAI Endorsed Transactions on Internet of Things. 2021;7: e1–e1.

[32] Zhang Y, Li X, Wang L, Fan S, Zhu L, Jiang S. An autocorrelation incremental fuzzy clustering framework based on dynamic conditional scoring model. Information Sciences. 2023;648: 119567.

